# Open surgical treatment for aortic arch and coronary artery aneurysms associated with immunoglobulin G4-related disease: a case report

**DOI:** 10.1186/s44215-023-00063-0

**Published:** 2023-08-14

**Authors:** Toshiki Yokoyama, Ryo Suzuki, Hiroshi Kurazumi, Akira Tanaka, Hiroo Kawano, Akihito Mikamo, Eiji Ikeda, Kimikazu Hamano

**Affiliations:** 1 Division of Cardiac Surgery, Department of Surgery and Clinical Science, Yamaguchi University Graduate School of Medicine, 1-1-1 Minami-Kogushi, Ube, Yamaguchi 755-8505 Japan; 2 Department of Pathology, Yamaguchi University Graduate School of Medicine, 1-1-1 Minami-Kogushi, Ube, Yamaguchi 755-8505 Japan; 3 Nursing and Laboratory Science Majors, Yamaguchi University Graduate School of Medicine, 1-1-1 Minami-Kogushi, Ube, Yamaguchi 755-8505 Japan

**Keywords:** Immunoglobulin G4-related disease; Aortic arch aneurysm, Coronary artery aneurysm, Coronary artery bypass grafting

## Abstract

**Background:**

Immunoglobulin G4-related disease (IgG4RD) is a systemic autoimmune disease characterized by a dense lymphoplasmacytic infiltrate containing IgG4-positive plasma cells that can cause aneurysms in various organs and tissues. There have been many reports of IgG4RD aneurysms; however, the surgical procedures adopted remain controversial. Herein, we report a case in which surgical treatment for IgG4-related aneurysms was successful.

**Case presentation:**

An 83-year-old woman with IgG4-related disease developed multiple aneurysms, including thoracic aortic aneurysm (TAA), right coronary artery aneurysm (CAA), abdominal aortic aneurysm, and common iliac artery aneurysm. Enhanced computed tomography (CT) revealed progressive enlargement of the TAA, while positron emission tomography-CT revealed intense ^18^F-fluorodeoxyglucose uptake around the TAA and CAA, suggesting the presence of adhesive tissue caused by adventitial inflammation around the aneurysms. Open aortic arch replacement with frozen elephant trunk procedure and coronary artery bypass grafting with ligation of the coronary artery were performed to treat the TAA and CAA. No surgical complications occurred. The postoperative CT scan showed no endoleak around the open stent graft and the coronary artery was patent. The patient is being followed up on an outpatient basis after discontinuing steroid therapy.

**Conclusions:**

We report the successful surgical treatment of IgG4-related multiple aneurysms. Our findings reveal that the main site of inflammation in aneurysms of IgG4RD is the adventitia and the frozen elephant trunk procedure is useful for avoiding complications.

## Background

Immunoglobulin G4-related disease (IgG4RD) is a systemic autoimmune disease characterized by a dense lymphoplasmacytic infiltrate containing IgG4-positive plasma cells that can cause aneurysms in various organs and tissues [1]. Steroid therapy and endovascular treatment are options for the treatment of aneurysms caused by IgG4RD. Herein, we report a case in which surgical treatment for IgG4RD aneurysms was successful.

## Case presentation

**Fig. 1:**
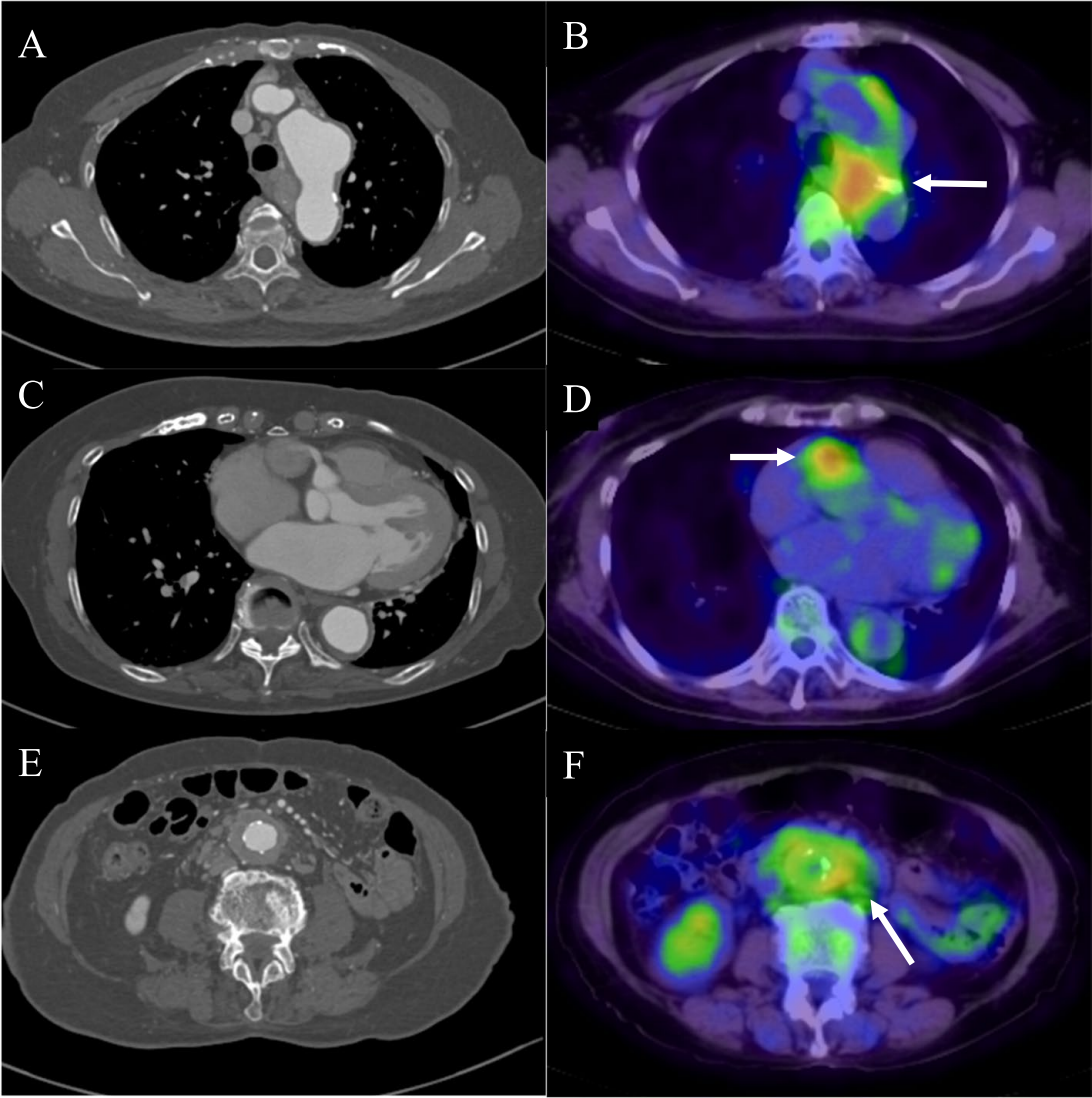
Preoperative enhanced computed tomography (CT) and positron emission tomography (PET)-CT images. Lesions with ^18^F-fluorodeoxyglucose (FDG) uptake are indicated by a white arrow. **A**, **B** Images of the thoracic aortic aneurysm (TAA) with a diameter of 50 mm. ^18^F-FDG uptake around the TAA (maximum standardized uptake value [SUVmax]: 5.6). **C**, **D** Images of the right coronary artery aneurysm (CAA) with a diameter of 21 mm. ^18^F-FDG uptake around the CAA (SUVmax: 5.1). **E**, **F** Images of abdominal aortic aneurysms with a diameter of 30 mm..^18^F-FDG uptake around the abdominal aortic aneurysm (SUVmax: 4.2)

**Fig. 2:**
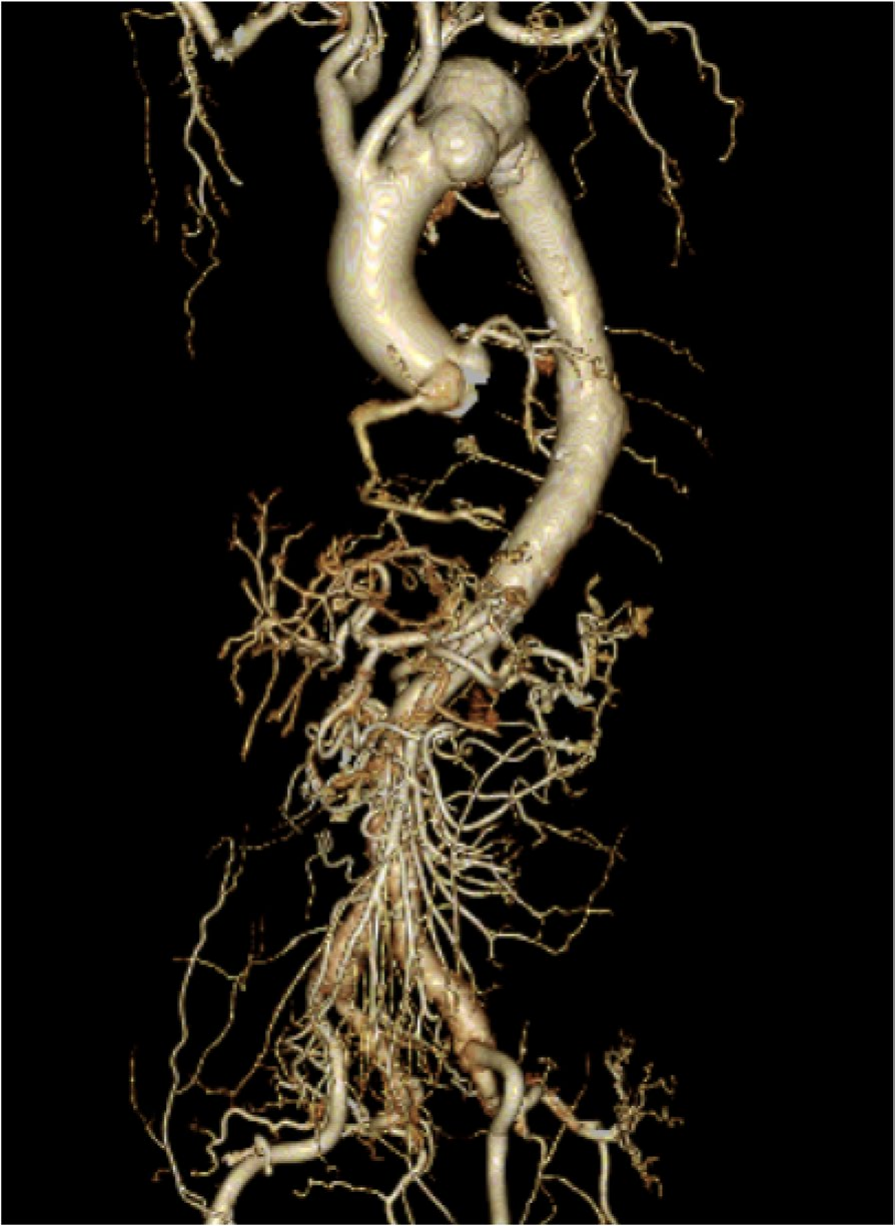
Preoperative 3-dimensional computed tomography (CT) of the aorta and coronary arteries

**Fig. 3:**
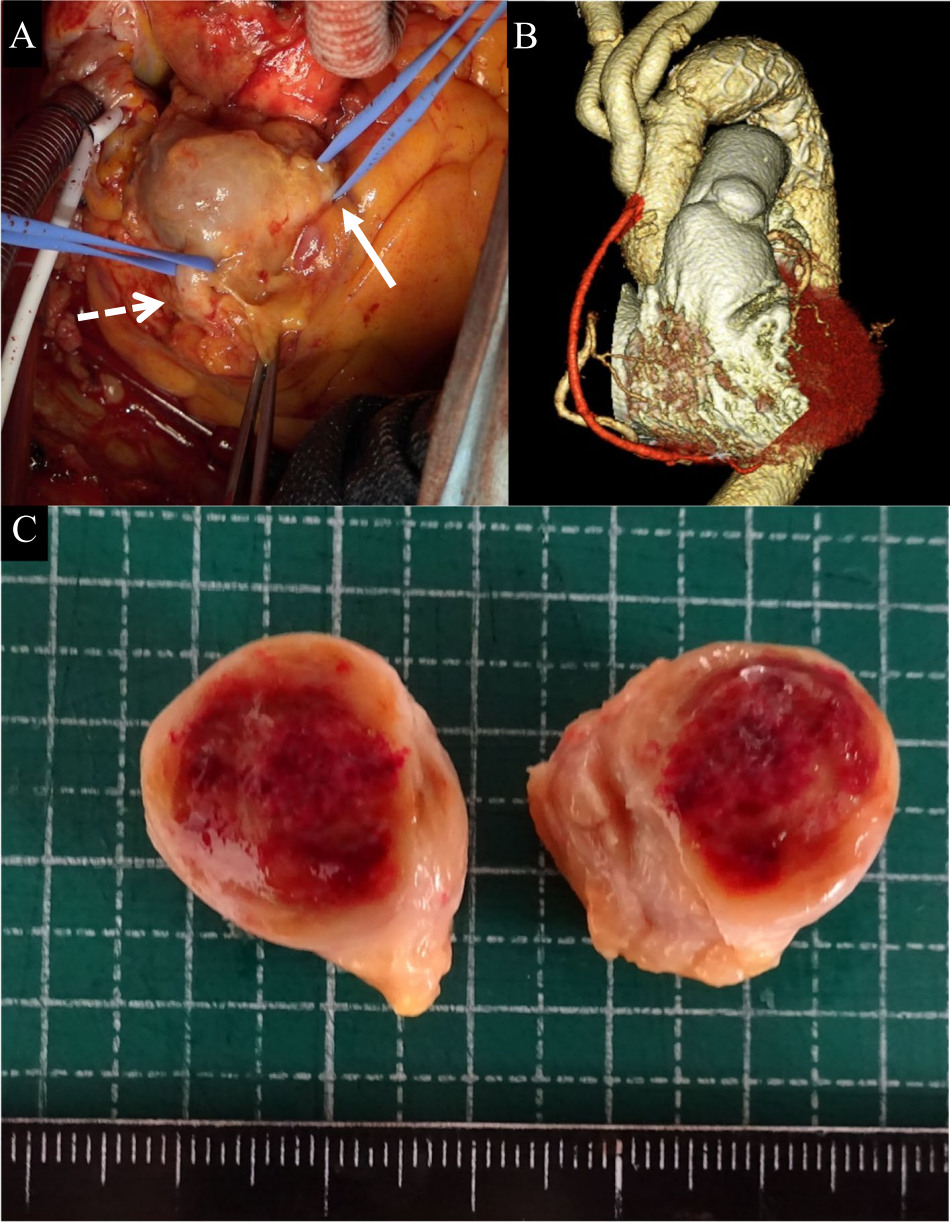
Intraoperative, macroscopic, and microscopic findings, postoperative computed tomography (CT) image, of the coronary artery aneurysm. **A** The inflow and outflow of the coronary aneurysm are encircled with white solid and dotted arrows, respectively. **B** Total aortic arch replacement, ligation of coronary artery aneurysm, and coronary artery bypass grafting (CABG). **C** The secant plane of the coronary artery aneurysm

An 83-year-old woman with IgG4RD developed multiple aneurysms, including thoracic aortic aneurysm (TAA), right coronary artery aneurysm (CAA), abdominal aortic aneurysm, and common iliac artery aneurysm. She was referred to our hospital for the examination of a high serum concentration of IgG4 (1610 mg/dL) around 7 years prior. Enhanced computed tomography (CT) revealed a cystic tumor of the appendix and multiple aneurysms of the right coronary artery, distal aortic arch, abdominal aorta, and common iliac arteries. Initially, she underwent appendicectomy for treatment of the cystic tumor. Further pathological examination revealed that IgG4-positive plasma cells were diffusely present in a specimen of the appendix with an IgG4/IgG ratio of > 40%. These clinical and histopathological findings led to a definitive diagnosis of IgG4RD. Since then, the aneurysms were monitored using CT every 6 months, and a gradual enlargement of the CAA was observed. Subsequently, the rapid growth of the TAA (≥ 5 mm in 6 months) was noted. Surgical treatment for TAA and CAA was performed at the age of 83 years. Preoperative blood examination results were as follows: white blood cell count, 5580 × 10^6^/L; eosinophils, 25.1%; C-reactive protein (CRP) level, 0.36 mg/dL; total protein level, 11.0 g/dL; gamma globulin fraction, 57.1%; serum IgG levels, 7011 mg/dL; and serum IgG4 levels, 3580 mg/dL. Preoperative positron emission tomography-CT (PET-CT) revealed an increased ^18^F-fluorodeoxyglucose (FDG) uptake around the TAA (maximum standardized uptake value [SUVmax] 5.6) and CAA (SUVmax 5.1), enhanced CT revealed a TAA with a diameter of 50 mm, and multidetector-row CT showed a CAA with a diameter of 21 mm (Fig. 1). A preoperative 3D-CT is shown in Fig. 2. Based on these images, the presence of adhesive tissue caused by inflammation around the aneurysms was suspected. The total arch replacement was performed using a 22-mm Gelweave four-branch graft (TERUMO, Tokyo, Japan) with stent graft insertion using a 29-mm Frozenix (Japan Lifeline, Tokyo, Japan) in the descending aorta (length of the stent portion was 90 mm). The total arch replacement was performed using circulatory arrest and selective cerebral perfusion at a rectal temperature of 23.1 °C. The total selective cerebral perfusion time and circulatory arrest time were 207 min and 83 min, respectively. The frozen elephant trunk procedure was selected to avoid the risk of intraoperative injury of adhesive lesions. Ligation of the inflow and outflow of the CAA (Fig. 3A), coronary artery bypass grafting (CABG), and aorto-coronary bypass to the posterior descending branch using a saphenous vein graft were also performed.

**Fig. 4:**
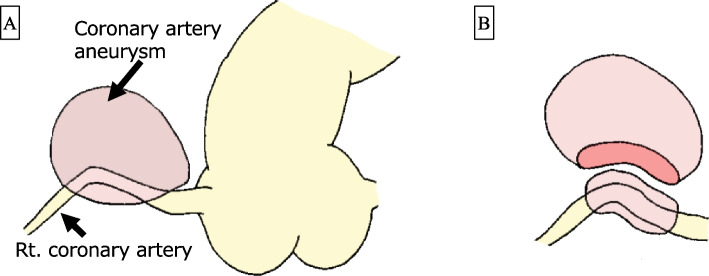
Schema of coronary artery aneurysm resection. **A** The right coronary artery aneurysm is presented as shown in the picture. **B** The coronary artery aneurism was resected from the right coronary artery as shown in the picture

**Fig. 5:**
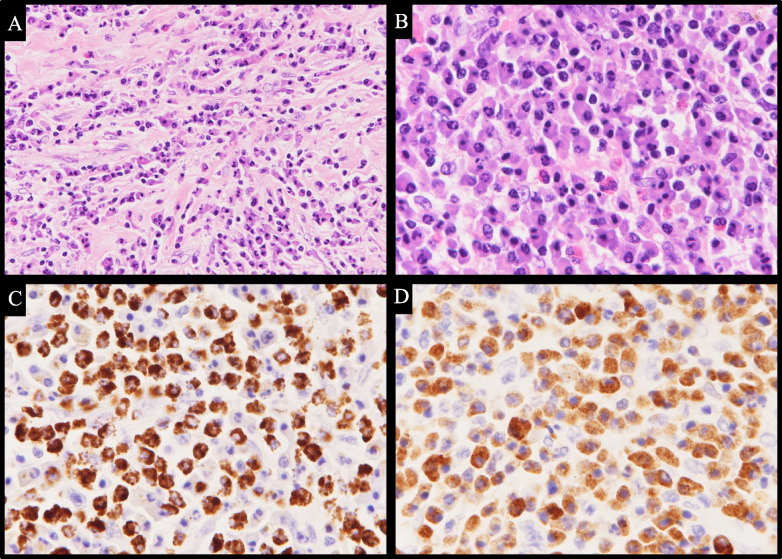
Pathological findings of coronary aneurysms. **A** Hematoxylin and eosin (HE) staining showing fibrosis (× 100). **B** HE staining showing infiltration of lymphocytes (× 200). **C** IgG staining for IgG-positive cells (× 200). **D** IgG4 staining showing more than 90% of plasma cells as IgG4-positive (× 200)

The intraoperative findings of the CAA and postoperative enhanced CT images are shown in Fig. 3B. A part of the CAA wall (Fig. 3C), which was a thickening of the adventitia, was resected as in Fig. 4 and examined pathologically. Histopathological sections of the CAA wall stained using hematoxylin and eosin (HE) showing fibrosis and IgG/G4 staining are shown in Fig. 5. The CAA specimen was surrounded by fibrous tissues with evident eosinophil and plasma cell infiltration (× 100 in Fig. 5A and × 200 in Fig. 5B), an IgG4/IgG-positive cell ratio of > 90% (IgG staining, × 200 in Fig. 5C, and IgG4 staining, × 200 in Fig. 5D), and no indication of obliterative phlebitis. The pathologic diagnosis was coronary artery adventitia owing to IgG4RD, and preoperative CT revealed that the severely thickened adventitia formed an aneurysm-like lesion.

There were no major sequelae in the postoperative clinical course of the patient. The patient was discharged 23 days after the surgery and regularly visits our outpatient clinic for follow-up examinations for the residual abdominal aortic aneurysm and common iliac artery aneurysm.

## Discussion and conclusions

IgG4RD is a fibroinflammatory disease first reported in Japan in 2001 [2]. It is characterized by dense lymphoid plasma cell infiltrates with a high rate of IgG4-positive plasma cells. Initially, IgG4RD was reported as pancreatitis. However, since then, an increasing number of cases have been reported in various organs such as the biliary system, salivary glands, periorbital tissues, kidneys, lungs, lymph nodes, meninges, aorta, breast, prostate, thyroid, pericardium, and skin, and IgG4RD was found to affect most organs [1]. The definitive diagnosis in this case was based on the following three findings: (1) clinically characteristic diffuse or localized enlargement, mass, nodule, or thickened lesion in a single organ or multiple organs; (2) hematological findings of hyper-IgG4emia (> 135 mg/dL); and (3) histopathological findings—(a) marked lymphocytic and plasma cell infiltration and fibrosis and (b) IgG4-positive plasma cell infiltrate, namely IgG4/IgG-positive cell ratio > 40% and IgG4-positive plasma cells > 10/high-power-field.

IgG4RD causes aneurysms and aortitis [3], and its lesions are often reported in multiple organs. Kasashima et al. [4] reported that this condition led to approximately 50% and 4% of inflammatory abdominal aneurysms and TAAs, respectively. General aortitis, except arteritis, related to IgG4RD is characterized by the damage and repair of endothelial and smooth muscle cells, while IgG4RD-related arteritis is characterized by its adventitia. In our case, a biopsied specimen of the adventitia of the CAA revealed a high level of infiltration of IgG4-positive plasma cells and preoperative PET-CT showed a strong adhesive lesion around the TAA, probably caused by adventitial inflammation. Tajima et al. [3] reported that perioperative complications and morbidity were higher after reconstruction for inflammatory aneurysms compared to those for atherosclerotic aneurysms because adhesive lesions around aneurysms make surgeries more difficult technically. The frozen elephant trunk procedure was, therefore, selected to avoid the risk of injury to these adhesive lesions, especially those of recurrent nerve injuries. Due to her advanced age, alternative procedures such as debranching thoracic endovascular aortic repair were considered. However, as her physical condition and preoperative examination were unremarkable, we concluded that she could undergo open surgery with cardiopulmonary bypass.

Steroid therapy for Takayasu arteritis and IgG4RD in symptomatic cases is recommended for patients in class I of the 2020 aortic disease management guidelines (JCS/JSCVS/JATS/JSVS 2020 Guidelines). However, a previous study reported that the risk of aneurysm rupture increases after steroid therapy as corticosteroids can reduce the inflammation of the adventitia, resulting in the thinning or weakening of the aneurysm itself [5]. Therefore, steroid therapy for aneurysms caused by IgG4RD is controversial, while surgery or endovascular therapy remains a reliable treatment method [6]. In our case, the patient was asymptomatic with respect to IgG4RD and the presence of the CAA alone was not an indication for operation. As the aortic diameter was 45 mm or less, we diagnosed the condition as enlargement of the aorta only; thus, steroid therapy was not administered preoperatively. However, as the rapid growth of the TAA (≥ 5 mm in 6 months) was noted, we decided to perform surgery before administering steroid hormones. Steroids were also not administered postoperatively because of residual aneurysms in the abdominal aorta and common iliac artery. Nishimura et al. reported that ascending aortic aneurysms and multiple coronary aneurysms in IgG4RD could be treated with steroid hormones [7]. To prevent rupture, Kamikawa et al. reported a hybrid treatment of IgG4RD CAA using endovascular therapy and coronary artery bypass grafting, even in patients receiving steroid hormones. Colombier et al. concluded that open surgery or medical treatment only could be controversial, because the aortic wall of IgG4RD aneurysms is thickened [8]. In our case, the aortic wall of the TAA was thin and enlarged; thus, we performed a combined operation for the TAA and CAA. To the best of our knowledge, this is the first report of open surgery for a combination of aortic arch and coronary aneurysms in a patient with IgG4RD.

In conclusion, preoperative PET-CT revealed multiple lesions with enhanced ^18^F-FDG uptake caused by IgG4RD in our patient. An open aortic arch replacement with stent graft insertion in the descending aorta and CABG with ligation of the coronary artery resulted in the successful treatment of the patient with no complications. Therefore, it is important to choose patient-specific approaches for reducing complications.

## Data Availability

Not applicable.

## Abbreviations

IgG4RD: Immunoglobulin G4-related disease
TAA: Thoracic aortic aneurysm
CAA: Coronary artery aneurysm
CT: Computed tomography
